# Multi-Frequency Magnetic Induction Tomography System and Algorithm for Imaging Metallic Objects

**DOI:** 10.3390/s21113671

**Published:** 2021-05-25

**Authors:** Gavin Dingley, Manuchehr Soleimani

**Affiliations:** Engineering Tomography Laboratory, Department of Electronic and Electrical Engineering, University of Bath, Claverton Down, Bath BA2 7AY, UK

**Keywords:** multi-frequency, magnetic induction tomography, hardware design

## Abstract

Magnetic induction tomography (MIT) is largely focused on applications in biomedical and industrial process engineering. MIT has a great potential for imaging metallic samples; however, there are fewer developments directed toward the testing and monitoring of metal components. Eddy-current non-destructive testing is well established, showing that corrosion, fatigue and mechanical loading are detectable in metals. Applying the same principles to MIT would provide a useful imaging tool for determining the condition of metal components. A compact MIT instrument is described, including the design aspects and system performance characterisation, assessing dynamic range and signal quality. The image rendering ability is assessed using both external and internal object inclusions. A multi-frequency MIT system has similar capabilities as transient based pulsed eddy current instruments. The forward model for frequency swap multi-frequency is solved, using a computationally efficient numerical modelling with the edge-based finite elements method. The image reconstruction for spectral imaging is done by adaptation of a spectrally correlative base algorithm, providing whole spectrum data for the conductivity or permeability.

## 1. Introduction

Research in the development of magnetic induction tomography (MIT) has largely concentrated on biomedical and industrial process engineering [1,2,3,4]. Studies directed toward biomedical applications often use saline low-conductivity samples to experimentally simulate tissue; these same methods are also applied to imaging pipeline flows [5,6]. Imaging of molten metal flow, and associated trapped gasses, are also investigated [7,8]. The industrial application of MIT in imaging materials with high-conductivity and high-permeability has not received as much attention [9]. Non-destructive testing and eddy-current testing (NDT and ECT, respectively) of metal components has been extensively investigated and successfully adopted as a tool in industry. However, in using ECT to render images for detecting corrosion [10,11,12,13,14], fatigue [15,16], defects [17,18,19] and physical loading [20,21,22], mechanical scanners are often employed [23,24,25,26,27,28]. Furthermore, while ECT is generally regarded as non-contact, close proximity sensor positioning is usually required. In this paper, a recently developed system is described for primarily investigating the application of MIT in metal component imaging. The described unit is designed for simplicity and compactness so that it is easily relocatable to industrial environments. Li et al. have used a similar system for studying defect measurements in hot steel [29]. In this first development, the system operates from 1 to 100 kHz and collects the magnitude of the perturbed excitation field only. A description of the system is given, along with the approach used to characterise a pre-existing sensor array to assess performance. Finally, initial results are presented, demonstrating the operation of the new MIT system. Studies have shown that it is possible, using alternating magnetic fields, to determine the condition of metal components non-destructively without physical contact. Corrosion, mechanical loading and surface discontinuities, such as cracks, are detectable using alternating fields from 1 to 100 kHz. However, ECT is generally the dominant method in this particular application, while MIT-based approaches have not received as much attention. Depending on the electromagnetic properties of the metal under test, the nature of the field perturbation differs. In general, magnetic materials, those with high permeability, enhance the excitation field by focusing the lines of flux, therefore leading to increased signal strength. Materials with high conductivity tend to attenuate the applied field due to the skin effect, resulting in reduced signal strength [30]. In metals, such as many steels, where the object of interest has both high-conductivity and high-permeability, the applied field tends to travel over the surface of the sample, a combination of both the skin effect and magnetic flux focusing [31,32]. Measurement of corrosion in metals is particularly directed toward assessment of steel rebars, which are encased in the concrete of free-standing structures, therefore making non-contact, non-destructive detection of particular interest. Lower frequencies, in the order of 10 to 100 Hz, are often preferred for their greater penetration, where absolute or differential eddy-current probes are often used [10,11,23]. However, there are also studies using higher frequency excitation fields, in the range of 1 to 100 kHz, but these are mostly based on the transient behaviour of coil probes [12,13,14,17,24,33,34]. Although penetration is small at higher frequencies, corrosion is generally a surface effect and therefore detectable [24]. Muttakin and Soleimani demonstrate metallic material characterisation using spatio-spectral analysis over a frequency range of 100 Hz to 100 kHz [35]. Complex impedance data from MIT array sensors are used to interrogate a variety of metallic structures, indicating a change in both phase and magnitude, in response to both sensor position and excitation field frequency. In both testing and monitoring of metal components, it is possible to indirectly measure mechanical stresses in high permeability materials. Ferrous metals exhibit a change in magnetic susceptibility when mechanically loaded (inverse magnetostrictive or Villari effect). While under physical deformation, the alignment of magnetic domains within the material grain structure tends to align with the axis of the applied force [20]. Ferrous metals with helical domains, caused by torsional stress, also show a change in magnetic susceptibility; the Matteucci effect [21]. Variations in stress are therefore reflected as changes in the inductance of coils magnetically coupled to the sample, or the coupling factor of transformer style sensors, as found in MIT systems [36,37,38]. Residual stress in ferrous materials, resulting from quenching and phase transformations, are also indirectly detectable using eddy-current methods [15,16,20,22]. Li et al. describe a method for evaluating the yield stress in cold rolled steel, where the sample is magnetically biased with a low-frequency magnetic field, driving the sample through its hysteresis loop, while recording the complex impedance of a 5 kHz ECT sensor coil [25]. Eddy-current crack detection in metal components, again using high-frequency excitation fields, has also been demonstrated. The abrupt discontinuity in surface conductivity, associated with cracks, interrupts the flow of induced currents [18,19,26,27,28,39,40,41]. Crack depth is reflected in the detected signal magnitude, but is also dependent upon sensor positioning relative to the sample (lift-off effect). Studies have shown that stepped excitation frequency can help indicate crack depth, potentially providing a way of mitigating the effects of sensor location [42,43,44].

## 2. Principles of MIT Fundamentals

The following is a description of the general principles used in the design and development of the MIT system, including the method of tomographic image reconstruction. Using magnitude only data, the instrument gives a visual indication of the metal component structure using frequency domain data. However, the coils used to form the sensor array have a frequency-dependent sensitivity. Furthermore, the mutual position and orientation of individual coil pairs also affects relative sensitivity; these two contributing factors affect the sample space dynamic range. To mitigate these effects, the system gain is adapted for the field frequency in use and the particular coil pair in operation during frame acquisition.

### 2.1. System Design

Assessing the electronics for the MIT system requires a characterisation of the scanning array, determining both the electrical and physical parameters, from which the required sensitivity and dynamic range is established. Current flowing in the excitation coil induces a voltage in the detector coil, where the sample object interacts with the intermediating field; therefore, the electromagnetic properties of the object are reflected in the induced voltage. The time harmonic expression for the voltage $v_{\det}$ induced in the detector coil of inductance $L_{\det}$, by a current $i_{\mathrm{ex}}$ flowing in the excitation coil, of inductance $L_{\mathrm{ex}}$, in the case of an object-free sample space, is given by:

(1) $$v_{\det}=-j\omega Mi_{\mathrm{ex}}$$

where *M* is the mutual inductance, which is a function of the magnetic coupling factor *k* between $L_{\det}$ and $L_{\mathrm{ex}}$, dependent upon the distance and relative angle between the two coils, given by $M=k\sqrt{L_{\det}L_{\mathrm{ex}}}$. However, the excitation coils and detector coils alternate their function during scanning and therefore are of the same inductance ($L_{\det}=L_{\mathrm{ex}}=L$), such that M=kL. Given that only magnitude information is used in the present system, Equation (1) becomes:

(2) $$|v_{\det}|=\omega kLi_{\mathrm{ex}}$$

From Equation (2), the maximum possible signal is determined by setting ω to the maximum system signal frequency $\omega_{\left(\max\right)}$ and *k* = 1, as 0 ≤k≤ 1 by definition. The maximum signal magnitude is usually dictated by the available hardware, for instance the maximum input voltage to the ADC before saturation ($V_{\mathrm{FS}}$), combined with initial amplification ($G_{\mathrm{int}}$) and tailoring of the excitation coil current $i_{\mathrm{ex}}$, therefore, from Equation (2):

(3) $$i_{\mathrm{ex}}=\frac{V_{\mathrm{FS}}}{G_{\mathrm{int}}\;\omega_{\left(\max\right)}\;L}$$

The voltage induced in the detector coil is dependent upon the excitation field frequency (ω) and coupling factor (*k*). So as to maintain a constant output signal magnitude, therefore maintaining the total numerical range, the system gain is configured for different coil pairs and excitation frequencies. The necessary additional system gain ($G_{\mathrm{sys}}$) is therefore given by:

(4) $$G_{\mathrm{sys}}\left(\omega ,k\right)=\left[\frac{\omega_{\left(\max\right)}}{\omega k}\right]$$

Setting the system gain to $G_{\mathrm{sys}}$, for a given coil pair coupling factor and frequency, while setting $i_{ex}$ to correspond with the ADC full-scale value (Equation (3)), ensures that the full dynamic range of the ADC is dedicated to capturing data related to the sample space.

### 2.2. Forward and Inverse Problems

In order to create MIT spectroscopic images, we need to simulate the measurement process in so-called forward modelling. The MIT forward model in each frequency can be described by means of eddy current models, and one can use the magnetic vector potential based formula, Equation (5).

(5) $$\nabla \times \frac{1}{\mu }\nabla \times A+j\omega \sigma A=J_{s}$$

where A is the magnetic vector potential, $J_{s}$ is the excitation current density, ω is the angular frequency, while σ and μ are the conductivity and permeability of the sample, respectively. The excitation current for each frequency is modelled in transmitting coils as the right-hand side of Equation (5). The measured induced voltage in receiving coils can be writtin as:

(6) $$V_{R}=-j\omega \int_{V}^{}A\;\cdot \;J_{0}dV$$

This gives a complex-value-induced voltage, however, as our system is based on amplitude, we use the amplitude of the voltage as the value returned from the forward model. The image reconstruction in MIT spectroscopy is a non-linear inverse problem, but to be able to solve this in a timely manner, a linearisation is used. This is done by calculation of the Jacobian or sensitivity matrix using the results of the forward problem. A sensitivity matrix showing the changes of induced voltage with the electrical conductivity within a given area in the imaging domain is described by, for an excitation coil *m* and detector coil *n*:

(7) $$\frac{\partial V_{mn}}{\partial \sigma }=\frac{-j\omega }{I_{m}I_{n}}\int_{\Omega }^{}E_{m}\;\cdot \;E_{n}d\Omega$$

While sensitivity to changes in permeability values can be described by:

(8) $$\frac{\partial V_{mn}}{\partial \mu }=\frac{-j\omega }{I_{m}I_{n}}\int_{\Omega }^{}H_{m}\;\cdot \;H_{n}d\Omega$$

We linearise the measured changes ΔV as a function of changes in conductivity or permeability, Δx; thus, the linear forward model can be written as:

(9) ΔV=JΔx

where J is the Jacobian, which is essentially a set of sensitivity distributions within the domain, which in this case is not square. The inverse MIT problem is also highly ill-posed, meaning it is sensitive to noise in measured data; in this case, regularisation methods are needed for a stable inverse solution. For the single frequency case, the Tikhonov Regularisation method is used to solve for Δx, as shown by Equations (10) and (11) [45]:

(10) Δx=SΔV

where the sensitivity matrix, S, is given by the Tikhonov Regularisation:

(11) $$S={\left(J^{T}J+\alpha^{2}\;I\right)}^{-1}J^{T}$$

where α is the regularisation parameter, usually determined as frequency-dependent, and *I* is the identity matrix. With a regularisation prior, which accounts for both spatial and spectral correlations between image elements. In spectral MIT, we have measured data in multiple frequencies, and the imaging results in neighbouring frequencies are similar. This allows for additional regulation in the frequency domain, for we can use a spectrally correlated algorithm, providing higher resolution and stability. Specifically, the data frame sequence is concatenated as $\tilde{\Delta V_{f}}=\left[\Delta V_{f-d};\ldots ;\Delta V_{f};\ldots ;\Delta V_{f+d}\right]$ and the resulting concatenated images $\tilde{\Delta \sigma_{f}}=\left[\Delta X_{f-d};\ldots ;\Delta X_{f};\ldots ;\Delta X_{f+d}\right]$. Therefore, the temporal inverse problem can be rewritten as [46]:

(12) $$\tilde{\Delta X_{f}}=\hat{S}\;\tilde{\Delta V_{f}}$$

For the multi-frequency case, calculating the sensitivity matrix S, using Equation (11) is computationally intensive; therefore, an alternative approach is used, where a sensitivity matrix, $\hat{S}$, representing the complete set of frequencies is used, providing a least squares fit approximation. In the following, Δx is a vector of 2500 elements, reshaped to a 50 by 50 image; however, an interpolation algorithm is used to smooth images. The quality of the rendered image depends on the sensitivity in the area of change, accuracy of measurement (SNR) and regularisation process. In the following sections, we attempt to link these relations for internal and external object imaging. For convenience, the frame data, ΔV, is normalised against the excitation frequency (ΔV/f), thus compensating for the array coil transfer function, as described by Equation (2).

## 3. MIT Hardware Design

In the following, details of the sensor array and system hardware operation are given. By using the analysis discussed in the previous section, the dynamic range of the instrument, as a function of frequency and array characteristics, is assessed.

### 3.1. Description of Array

**Figure 1:**
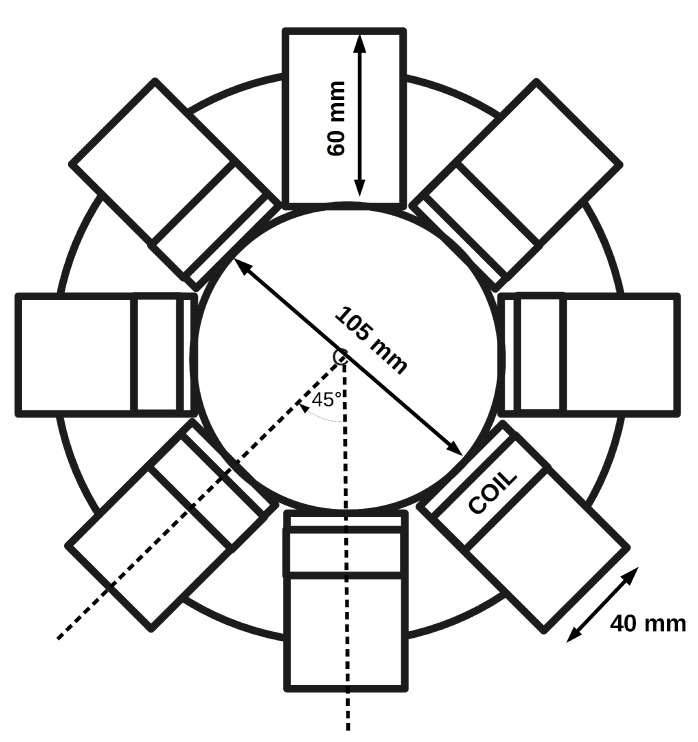
Diagram of array, indicating coil position and dimensions.

The array is formed from eight coils positioned to form a 105 mm diameter circular sample space, with a coil angular displacement of 45${}^{\circ }$, as shown in Figure 1. Each coil central axis is positioned vertically 50 mm from the base of the sample space, the combined height of the base mount material and connecting cables.

Each coil has a diameter of 40 mm, wound with 55 turns of 32 swg (0.274 mm diam.) enamelled copper wire, giving a coil length of 15 mm; the axis coil centre to opposite coil centre distance is 140 mm. The measurement of the coils with an LCR instrument gives an average inductance of 126.97 μH (±0.55) and an average equivalent series resistance of 2.61 Ω (±0.49). Free space coupling between the coils, where the sample space is empty, was measured for each of the four angular coil pair displacements, as indicated in Figure 1, resulting in eight individual measurements for 45${}^{\circ }$, 90${}^{\circ }$, 135${}^{\circ }$, and four for 180${}^{\circ }$. Table 1 summarises the average angular coupling factors and standard deviation.

**Figure 2:**
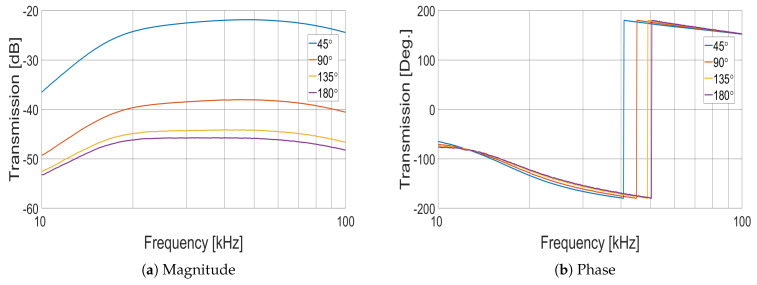
Coil pair transmission measurements ($S_{21}$), for the four different angular displacements.

Measuring the transmission between coil pairs is another method of characterising the array. Using a vector network analyser (VNA), $S_{21}$ phase and magnitude measurements were taken over 928 frequency points from 10 to 100 kHz. Phase and magnitude data for each coil pair angular displacement are shown in Figure 2. At the lower frequencies, the magnitude follows the trend given by Equation (2), while at the higher frequencies, the impedance of the excitation coil becomes apparent. The coil pair phase data in Figure 2 shows distinct phase inflections from approximately 40 to 50 kHz, depending on the particular coil pair angular displacement, suggesting a resonance at a frequency dependent upon the coupling factor. Impedance measurements of a single coil, derived from VNA $S_{11}$ reflection parameters, show a resonance occurring at 1 MHz; however, over the 1 to 100 kHz frequency range of interest, a single coil acts as a pure inductance.

**Figure 3:**
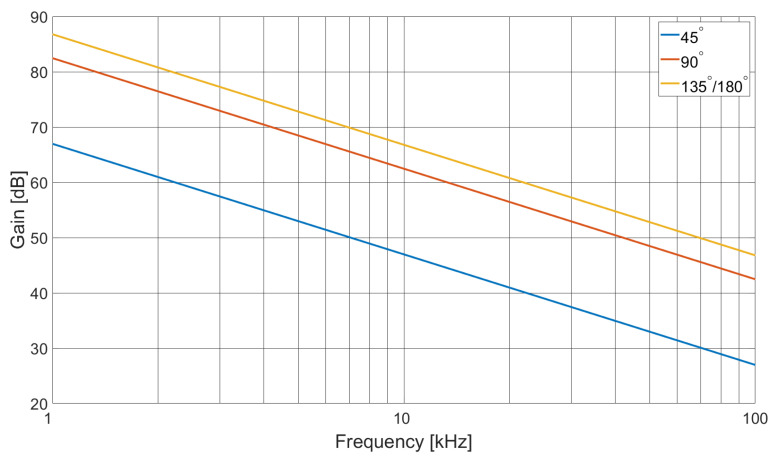
Graph showing required system gain as a function of frequency for different coil pair angles.

From the array data given in Table 1, the required system gain, as a function of the coil pair angular displacement and excitation field frequency, is calculated with Equation (4), the result of which is shown graphically in Figure 3.

### 3.2. Hardware Description

**Figure 4:**
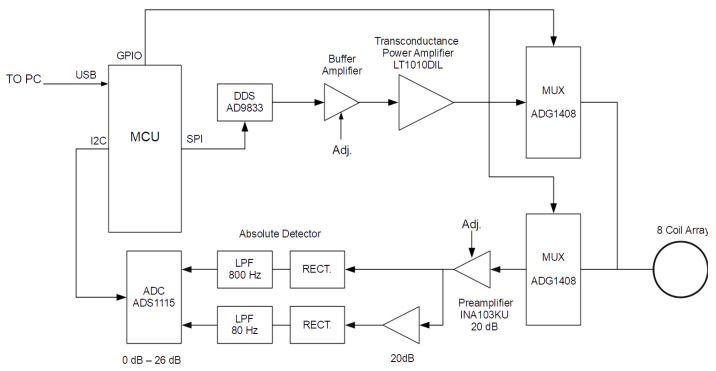
System diagram of the MIT instrument.

Testing of metal samples in an industrial environment requires a physically compact system that is easily deployed; therefore, the hardware was constructed as a single stand-alone unit. System configuration, control and data acquisition were performed by an AVR 8-bit microcontroller (MCU), which communicates with a host PC over a USB bridge, as indicated in the system diagram Figure 4.

During the scanning operation, one coil is selected as the excitation source, while the other coils are multiplexed in sequence as the detector coil. There are two ADG1408 multiplexers, one of which selects the excitation coil, while the other selects the detector; both are controlled by the MCU GPIO port. The common signal input to the excitation multiplexer is fed with the current from a transconductance amplifier, built around an LT1010 power op-amp, set to output a current of 100 mA(rms). An AD9833 direct digital synthesizer (DDS) generates the 1 to 100 kHz tone that is fed to the transconductance amplifier, via a buffer stage to condition and set the sinusoidal signal amplitude. Tone frequency is set by the MCU over an SPI connection to the DDS, while the current amplitude is adjusted manually. The common signal output of the receiver multiplexer is fed to a coil pre-amplifier, which has a manually adjustable pre-set gain of nominally 22 dB and provides the initial gain ($G_{\mathrm{int}}$). Signal integrity is maintained by employing separate power and signal grounds connected at a single point. All coils are connected to the power ground plane, therefore providing a clear current return when set as the transmitter. The coil pre-amplifier is built around a Burr-Brown INA103KU instrumentation amplifier; therefore, the differential input is used to re-reference the amplifier output to the signal ground. Op-amp based precision rectifiers, incorporating a low-pass filter network (LPF), are used to convert the AC magnitude of the received signal into a DC voltage. Given the operating frequency range, the LPF of a single detector stage is not sufficient to provide both low-ripple voltage and fast settling time, therefore two selectable stages are used. Given the 40 dB sensitivity range of the detector coil over the total frequency span, a 20 dB amplifier stage precedes the lower frequency absolute detector. The absolute detector DC voltage level outputs are digitised by an ADS1115 ADC, where two of the four multiplexed analogue inputs are used. The ADS1115 also contains a programmable amplifier, with gain settings of effectively 0, 2, 8, 14, 20 and 26 dB, configured by the MCU over an I2C bus. Selecting the appropriate analogue channel of the ADC, for the frequency range of interest, while setting the ADS1115 gain, the effects of coil frequency response and coil pair coupling are compensated, therefore dedicating the dynamic range of the ADC to capturing sample space data. A total system gain of 20 to 66 dB is therefore available; however, from Figure 3, it is apparent that the upper gain is deficient by 21 dB, and this reduction in sensitivity will impact the dynamic range dedicated to the sample space. The ADS1115 has a 16 bit ADC; however, this is for bipolar operation, so only 15 bits are used in the present application, resulting in a total dynamic range of approximately 90 dB (6.02$\;\times \;N_{\mathrm{bits}}$). However, as a result of the deficiency in system gain, 21 dB of the ADC dynamic range is allocated to compensating for array detector coil sensitivity; therefore, 69 dB remains for the sample space.

## 4. Results and Discussion

At present, the system has a frame acquisition time of 750 ms, partly attributed to the 10 ms dwell time of the absolute detector, therefore contributing 280 ms to the total acquisition period, i.e., 10 ms ×N(N−1)/2, where *N* is the number of coils (in the present case, eight). Serial communications and delays resulting from PC activity contribute the remaining 470 ms. Absolute detector dwell times are directly related to the field excitation frequency; in the present system, this is fixed to 10 ms to cater for the lowest expected frequency of 1 kHz. A bank of absolute detectors tailored for sections of the total frequency range, in terms of amplification and dwell time would improve both dynamic range and frame acquisition time. To assess the performance of the system, measurements were taken to determine the quality of frame data and resulting reconstructed images.

### 4.1. SNR Measurements of Frame Data

**Figure 5:**
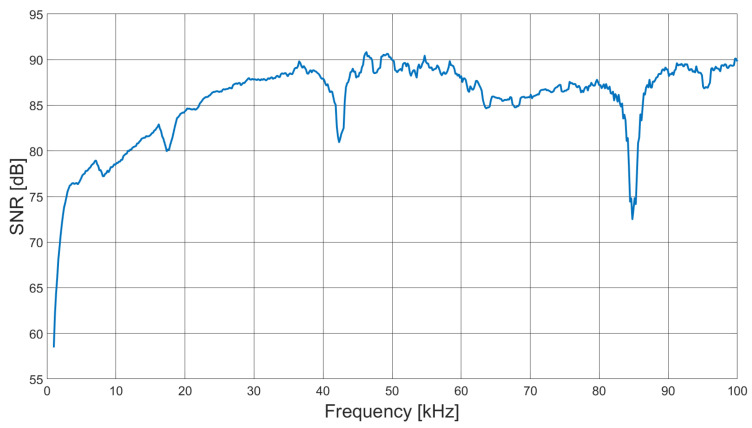
SNR as a function of excitation field frequency.

Figure 5 shows the signal to noise ratio (SNR) as a function of field excitation frequency. A total of 100 sets of frame data were taken to determine the mean and standard deviation for each coil pair, from which the average SNR over a frame was taken for each frequency point. A total of 600 frequency points were taken from 1 to 100 kHz—the nominal operating range of the system.

**Figure 6:**
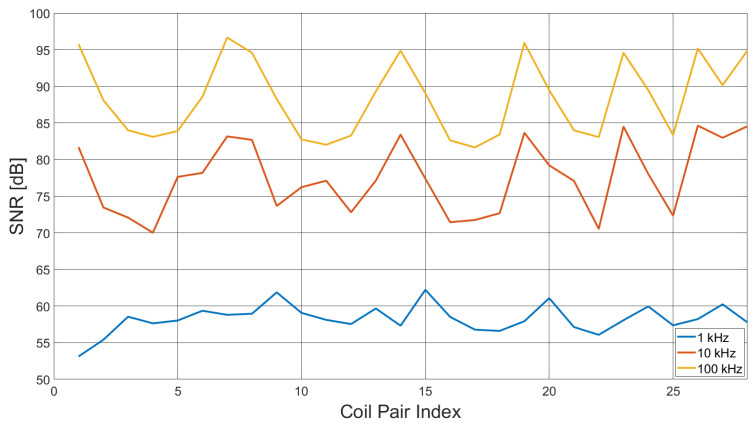
Frame data SNR for excitation field frequencies 1, 10 and 100 kHz.

Two artefacts in Figure 5 are apparent; first, a notch at 42 kHz with a depth of 6 dB, which corresponds with the phase inflection in Figure 2b, identified as a resonance. Although small, the resonance creates a local instability in the frequency sweep, resulting in slightly decreased SNR. A second larger artefact in Figure 5 occurs at 84 kHz, with a decrease in SNR of approximately 14 dB. On further investigation, it was found that at this frequency, the excitation field increases by 6 dB, inducing an instability in the transconductance amplifier, therefore decreasing SNR. The harmonic relation between the smaller artefact at 42 kHz and the larger decrease in SNR at 84 kHz suggests that the larger artefact results from the interaction of the transconductance amplifier with the coil pair resonance. Figure 6 shows the measured SNR over a single frame, i.e., SNR as a function of the coil pair, for 1, 10 and 100 kHz excitation fields.

From Figure 6, it is apparent that the SNR is dependent upon the coil pair, which directly corresponds with the coupling factor. However, the frequency of the excitation field has a greater influence, as also indicated by Figure 5.

### 4.2. Measurements of Reconstructed Images

System performance measurements based on reconstructed images from frame data were made using the algorithms described by Adler et al. [47]. Of particular interest are the relative resolution and relative image deformation with frequency, particularly when other conducting objects are introduced into the sample space with the sample of interest. Quantitative assessment of image reconstruction begins with determining the optimum amplitude threshold of the column vector $\hat{x}$ representing the reconstructed image. As the objects are clearly defined and known, the threshold value is determined empirically and defined by a factor $k_{th}$, where $1\geq k_{th}\geq 0$, resulting in the amplitude-limited vector ${\hat{x}}_{q}$, from Equation (13):

(13) $${\left[{\hat{x}}_{q}\right]}_{i}=\left\{\begin{array}{cc} 1 & \mathrm{if}\;{\left[\hat{x}\right]}_{i}\geq k_{th}\;\cdot \;\max\left(\hat{x}\right)\\ 0 & \mathrm{otherwise} \end{array}\right.$$

Image resolution (RES) is determined by the area ratio of the amplitude-limited image, represented by vector ${\hat{x}}_{q}$ and the total image area ($A_{0}$), measured in pixels; therefore, it has a value 1≥RES≥0. A square root is used to represent this ratio in terms of radius; see Equation (14):

(14) $$\mathrm{RES}=\sqrt{\frac{\sum_{k}{\left[{\hat{x}}_{q}\right]}_{k}}{A_{0}}}$$

Shape deformation (SD) is defined as the fraction of the threshold-limited, reconstructed image ($\left[{\hat{x}}_{q}\right]$) outside a circular boundary of equal area; therefore, it is a ratio with a value 1≥SD≥0, given by Equation (15):

(15) $$\mathrm{SD}=\frac{\sum_{k\neq C}{\left[{\hat{x}}_{q}\right]}_{k}}{\sum_{k}{\left[{\hat{x}}_{q}\right]}_{k}}$$

**Figure 7:**
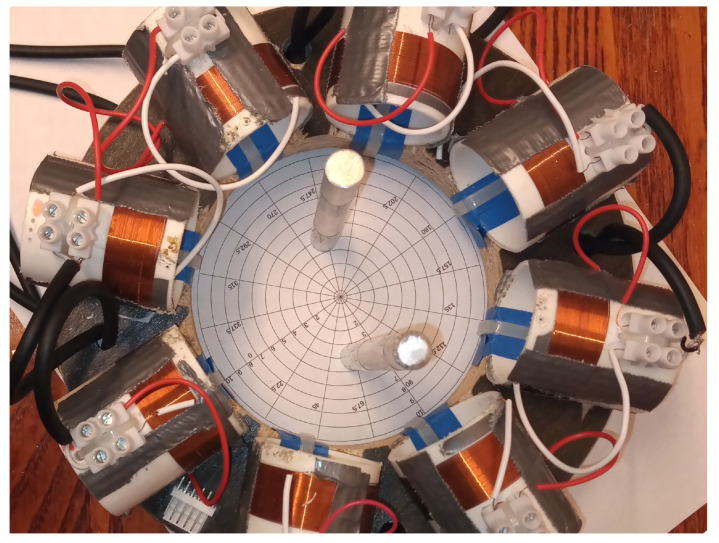
Photograph of inclusion measurements using aluminium rod samples 10 mm in diameter and 100 mm in length, with a 105 mm diameter circular sample space.

**Figure 8:**
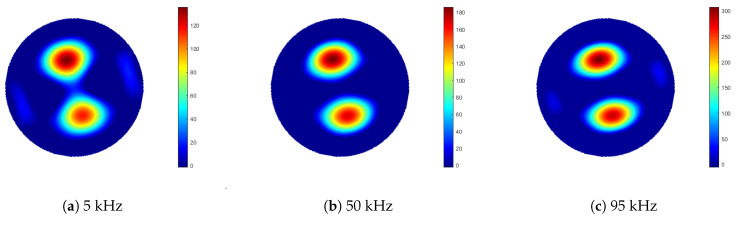
Reconstructed images from aluminium external object inclusion measurements ($\Delta V=V_{\mathrm{both}}-V_{\mathrm{bg}}$), acquired using 5, 50 and 95 kHz excitation field frequencies. Images represent a circular sample space of 105 mm diameter.

**Figure 9:**
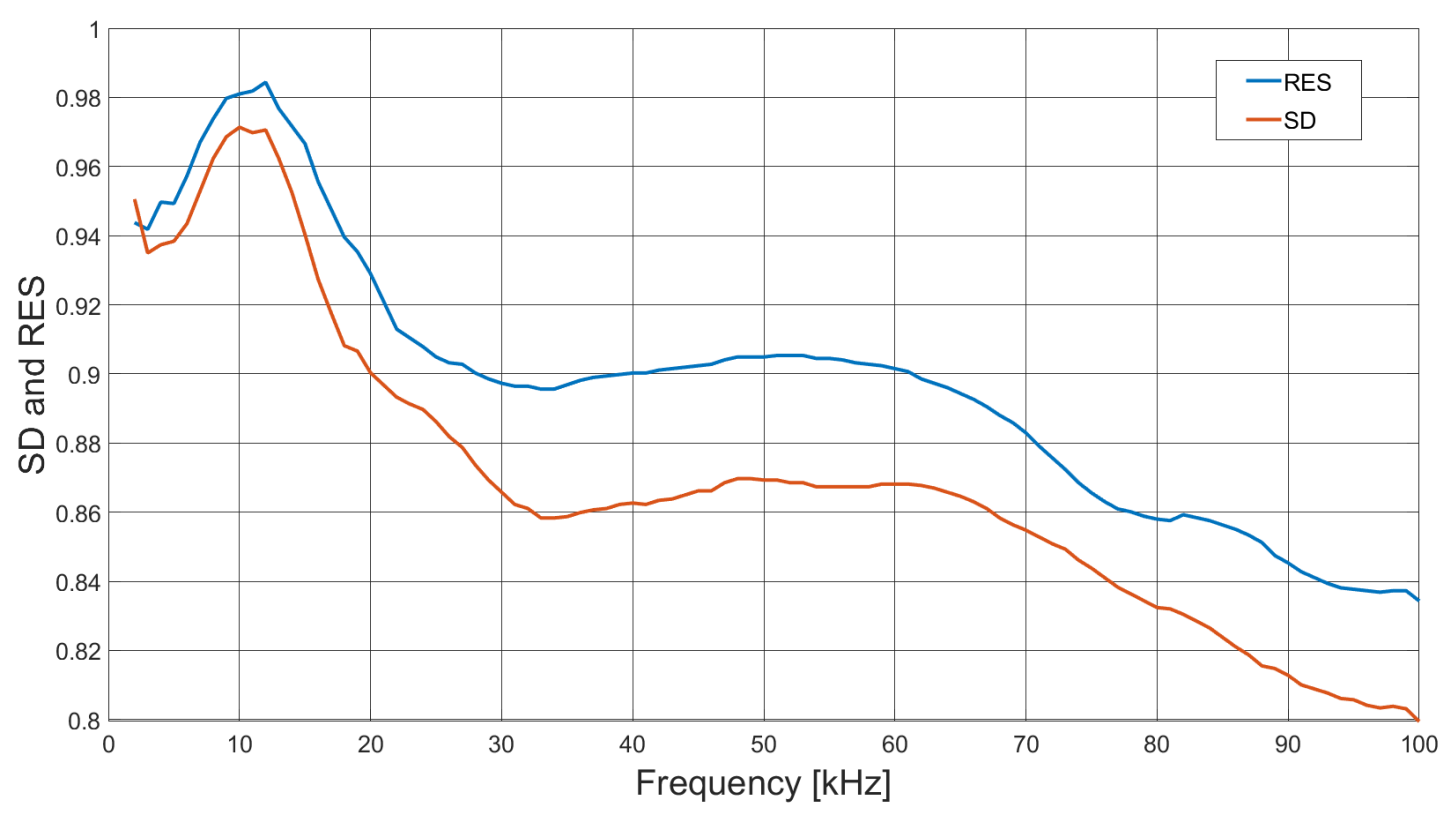
Reconstructed image resolution (RES) and shape deformation (SD) of dual aluminium external object inclusion data, applying a threshold value of $k_{th}$ = 0.3 in Equations (13)–(15), as a function of excitation field frequency 2 to 100 kHz.

Measurements were taken of an aluminium rod sample, 100 mm in length and 10 mm in diameter. In the first set of measurements, the sample rod was positioned at a radius of 40 mm from the centre of the sample space. An identical rod was then positioned symmetrically opposite the sample, located at an equal radial distance from the sample space centre, see Figure 7. Data were collected for 100 frequency points, over a range of 1 to 100 kHz. Background data, where the sample space is empty, were taken by averaging 100 frames ($V_{\mathrm{bg}}$). With the rods positioned in the sample space, each frequency point was averaged over five frames of data ($V_{\mathrm{both}}$), from which the background data were subtracted ($\Delta V=V_{\mathrm{both}}-V_{\mathrm{bg}}$). Images were reconstructed using Tikhonov regularisation, as described previously in Equation (11), then the threshold condition was applied, as described by Equation (13). It is noted that the regularisation parameter α in Equation (11) should be frequency dependent; however, in this case, the same value was used across the frequency range. Figure 8 shows the reconstructed images for 5, 50 and 95 kHz. From Equations (14) and (15), the resolution and shape deformation of the reconstructed image are calculated for each frequency point, as shown in Figure 9.

**Figure 10:**
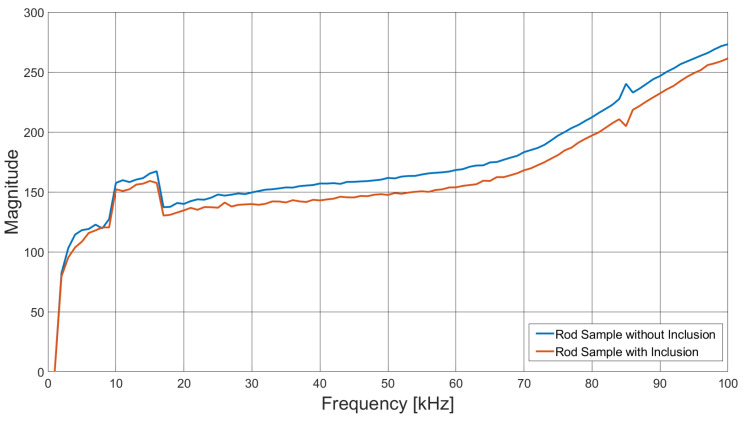
Image maximum (max(Δx)) as a function of frequency, for the rendered images of the aluminium rod sample individually ($\Delta V=V_{\mathrm{sgl}}-V_{\mathrm{bg}}$) and with the including object ($\Delta V=V_{\mathrm{both}}-V_{\mathrm{sgl}}$).

Figure 10 shows the variation in image maximum (max(Δx)) as a function of excitation field frequency for both the single rod in the sample space ($\Delta V=V_{\mathrm{sgl}}-V_{\mathrm{bg}}$) and with the addition of the identical external rod ($\Delta V=V_{\mathrm{both}}-V_{\mathrm{sgl}}$).

Figure 8 suggests that there is very little variation in image quality with field excitation frequency, although Figure 8a indicates some degradation at the lower frequencies, caused by lower SNR; see also Figure 5. Figure 9 gives a better indication of the image quality as a function of frequency, where below 20 kHz, both the resolution and deformation are relatively poor; however, they improve with increased field frequency, again following the SNR trend in Figure 5. There is a slight degradation in resolution as a result of the previously identified 84 kHz resonance, but this is a direct consequence of the associated decrease in SNR. Figure 10 indicates that there is very little difference in the image maximum with or without the inclusion of an identical rod sample in the sample space, which corresponds with the image quality observations drawn from Figure 8. Resonance effects are apparent, and a system frequency response below approximately 10 kHz is clearly shown, resulting in the lower SNR and, hence, image quality at these frequencies.

**Figure 11:**
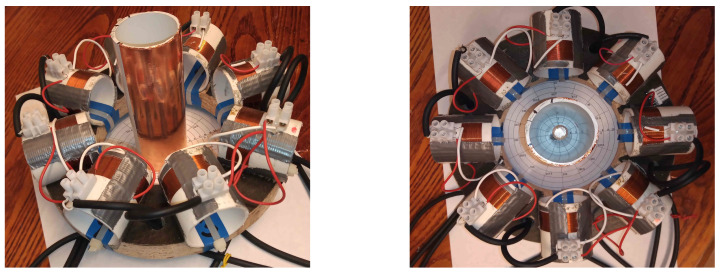
Photographs of internal object measurements using an aluminium rod and copper shroud.

**Figure 12:**
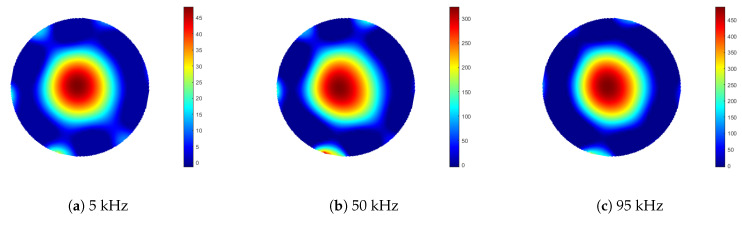
Reconstructed image measurements of aluminium rod partially concealed in a copper shroud, acquired using 5, 50 and 95 kHz excitation field frequencies. Image constructed from frame data of both rod and shroud present in the sample space ($\Delta V=V_{\mathrm{both}}-V_{\mathrm{bg}}$).

**Figure 13:**
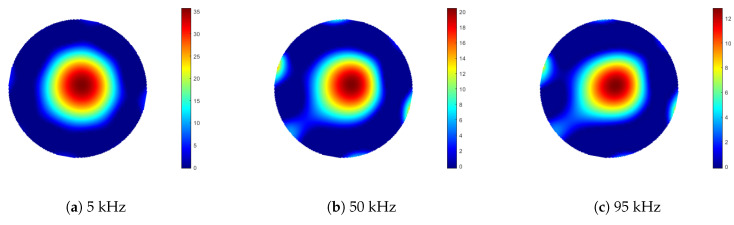
Reconstructed image of aluminium rod partially encapsulated in a copper shroud, with shroud frame data subtracted ($\Delta V=V_{\mathrm{both}}-V_{\mathrm{shrd}}$), leaving the rod; acquired using 5, 50 and 95 kHz excitation field frequencies. Image (**a**) clearly shows the aluminium rod, while the influence of the shroud at higher frequencies is apparent in (**b**,**c**), where the rod is weaker and distorted.

**Figure 14:**
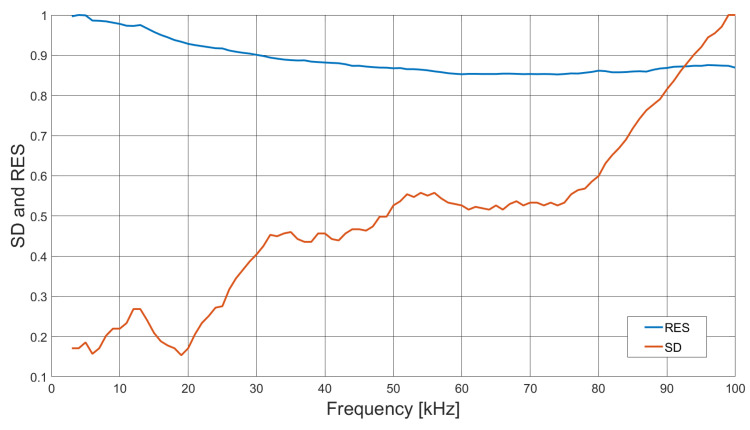
Reconstructed image resolution (RES) and shape deformation (SD) of internal object measurement using a conducting shroud, where shroud frame data are subtracted ($\Delta V=V_{\mathrm{both}}-V_{\mathrm{shrd}}$). Using a threshold value of $k_{th}$ = 0.3 in Equations (13)–(15), frame data were collected over a frequency range of 2 to 100 kHz.

Reconstructed concealed object image measurements were also taken of a partially enclosed aluminium rod, centrally positioned within a C-shaped cylindrical shroud, 42 mm in diameter and with a height of 110 mm; Figure 11 shows the arrangement. The conducting material forming the shroud is 40 μm thick copper, so minimal attenuation was provided to the applied excitation field. Both the rod and shroud were placed in the centre of the sample space, where again 100 frequency points were recorded, each averaged over five frames. Reconstructed images with the rod and shroud present are shown in Figure 12, where the background data are subtracted from the frame data ($\Delta V=V_{\mathrm{both}}-V_{\mathrm{bg}}$). Figure 13 is reconstructed from the frame data with the rod and shroud in position, but with the frame data of the shroud subtracted ($\Delta V=V_{\mathrm{both}}-V_{\mathrm{shrd}}$), thus leaving the rod. Figure 14 shows plots of both the resolution (RES) and shape deformation (SD) as a function of frequency, derived from the images in Figure 13 using Equations (13)–(15), i.e., the data from both rod and shroud, with the data from the shroud subtracted ($\Delta V=V_{\mathrm{both}}-V_{\mathrm{shrd}}$).

**Figure 15:**
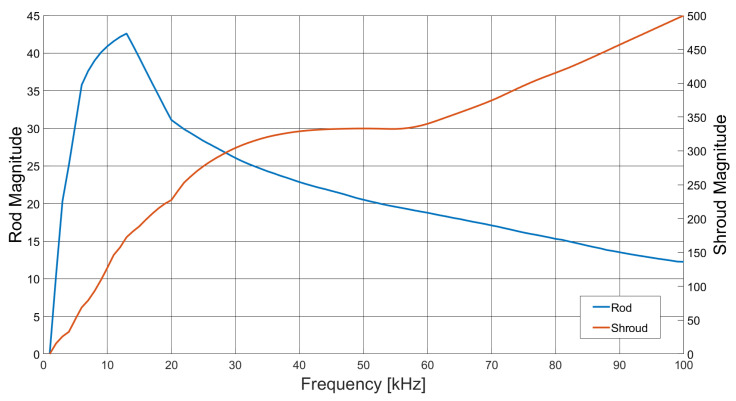
Image maximum as a function of frequency, for the rendered images for both the aluminium rod sample and the internally-included copper shroud independently.

Figure 15 shows the variation in image maximum (max(Δx)) as a function of frequency for both the rod ($\Delta V=V_{\mathrm{both}}-V_{\mathrm{shrd}}$) and shroud ($\Delta V=V_{\mathrm{shrd}}-V_{\mathrm{bg}}$). With increasing excitation field frequency, the internally-included shroud has increased visibility as a result of the skin effect, therefore decreasing the visibility of the rod by its shielding action.

Figure 12 indicates that there is little variation in the reconstructed image with frequency; however, the shroud is dominant and hides the image of the rod. Subtracting the frame data of the shroud alone, without the rod, reveals the internal object, as shown in Figure 13. It is apparent from Figure 13 that at higher field frequencies the skin effect of the shroud shields the rod, thus attenuating the image. Figure 15 further indicates how the presence of the shroud in rendered images is more dominant at higher frequencies, while that of the sample rod is reduced. Below approximately 10 kHz, the frequency response is low, as a result of the limitations of the system; however, beyond this point, the rod has a strong presence in the rendered image, but rapidly decreases with frequency, as it is shielded by the shroud. Figure 14 shows the resolution and shape deformation of the reconstructed images in Figure 13 as a function of frequency, which indicates that the rod image resolution improves slightly with increased frequency, while the shape deformation degrades. Increased resolution results from the improved SNR with frequency, as indicated by Figure 5, while the shape deformation is dependent upon the increasing influence of the conducting shroud with frequency, due to the skin effect.

### 4.3. Discussion of Results

Resolution and shape deformation are dependent upon frame data SNR, where any decrease in signal or increase in noise results in diminished performance. Reduction in coil pair transmission, from either low excitation frequency, coil coupling, or the presence of shielding, decreases signal strength. Although a lower excitation frequency corresponds with a smaller signal induced in the detector coil, it also provides greater shield penetration, thus presenting a trade-off. Noise primarily originates from solid-state devices in detector amplifiers in the form of Gaussian noise. However, the excitation coil driver, in this case, the transconductance amplifier, can also introduce noise in the form of instabilities. Self-resonance of array coils can cause instability noise when either acts as a detector or field generator; in the present case, the latter is most apparent. Although characterisation of an array provides insight into the dynamic-range dedicated to the sample space, frequency domain measurements of coil impedance and coil pair transmission are also important, indicating the presence of excitation frequencies offering reduced performance.

## 5. Conclusions

Although the application of MIT for metal component testing is growing, the methods described for eddy-current testing suggest the potential for generating cross-sectional images indicating corrosion and fatigue. A compact magnitude-only MIT system was developed for investigating metal component imaging in industrial test facilities. Given the levels of excitation-field perturbation over a range of metals, having either high conductivity, high permeability or a combination of both, a significant dynamic range is needed, which was analytically quantified for a given sensor array. The ability to alter the excitation field frequency opens the possibility of spectral based image reconstruction, as well as greater image rendering stability, for which an algorithm was presented. Initial test results were given indicating the performance of the new system, particularly in discriminating metallic samples within the sample space. While image quality improves with frequency for external objects, there is a degradation in image quality for concealed objects. Characterisation of a chosen array not only gives an indication of required system gain, but also identifies the presence of parasitic resonances that can potentially degrade rendered image quality.

## Figures and Tables

**Table 1:** Average coupling factors of coil pairs with the four angular displacements.

Angle	Average *k*	STD *k*
45°	0.044	0.0037
90°	0.0075	0.0029
135°	0.0046	0.0023
180°	0.0046	0.00218
